# In Vivo Neural Recording and Electrochemical Performance of Microelectrode Arrays Modified by Rough-Surfaced AuPt Alloy Nanoparticles with Nanoporosity

**DOI:** 10.3390/s16111851

**Published:** 2016-11-03

**Authors:** Zongya Zhao, Ruxue Gong, Liang Zheng, Jue Wang

**Affiliations:** 1 Key Laboratory of Biomedical Information Engineering of the Ministry of Education, Institute of Biomedical Engineering, School of Life Science and Technology, Xi’an Jiaotong University, Xi’an 710049, China; zhaozongya2010@stu.xjtu.edu.cn (Z.Z.); gongruxue@stu.xjtu.edu.cn (R.G.); ylzhengliang@stu.xjtu.edu.cn (L.Z.); 2 National Engineering Research Center of Health Care and Medical Devices, Xi’an Jiaotong University Branch, Xi’an 710049, China

**Keywords:** rough-surfaced AuPt alloy nanoparticles, in vivo neural recording, electro-co-deposition, microelectrode arrays

## Abstract

In order to reduce the impedance and improve in vivo neural recording performance of our developed Michigan type silicon electrodes, rough-surfaced AuPt alloy nanoparticles with nanoporosity were deposited on gold microelectrode sites through electro-co-deposition of Au-Pt-Cu alloy nanoparticles, followed by chemical dealloying Cu. The AuPt alloy nanoparticles modified gold microelectrode sites were characterized by scanning electron microscopy (SEM), electrochemical impedance spectroscopy (EIS), cyclic voltammetry (CV) and in vivo neural recording experiment. The SEM images showed that the prepared AuPt alloy nanoparticles exhibited cauliflower-like shapes and possessed very rough surfaces with many different sizes of pores. Average impedance of rough-surfaced AuPt alloy nanoparticles modified sites was 0.23 MΩ at 1 kHz, which was only 4.7% of that of bare gold microelectrode sites (4.9 MΩ), and corresponding in vitro background noise in the range of 1 Hz to 7500 Hz decreased to 7.5 $\mu V_{\mathrm{rms}}$ from 34.1 $\mu V_{\mathrm{rms}}$ at bare gold microelectrode sites. Spontaneous spike signal recording was used to evaluate in vivo neural recording performance of modified microelectrode sites, and results showed that rough-surfaced AuPt alloy nanoparticles modified microelectrode sites exhibited higher average spike signal-to-noise ratio (SNR) of 4.8 in lateral globus pallidus (GPe) due to lower background noise compared to control microelectrodes. Electro-co-deposition of Au-Pt-Cu alloy nanoparticles combined with chemical dealloying Cu was a convenient way for increasing the effective surface area of microelectrode sites, which could reduce electrode impedance and improve the quality of in vivo spike signal recording.

## 1. Introduction

In vivo neural recording provides a useful method for neuroscientists to understand the basic organization and operation of the nervous system, and especially the electrophysiological changes caused by neurological disorders such as Parkinson’s disease [1,2,3,4,5] and Alzheimer’s disease [6]. Although microwire-based electrodes have been a versatile tool for neuroscientists for decades, the limitations of microwire-based electrodes, such as difficult implantation, low spatial resolution, low reproducibility and manual construction are also well known [7,8]. The rapid development of micro-electro-mechanical systems (MEMS) technology in the biomedical field provides a feasible solution for the above-mentioned problems. With MEMS technology, the precise definition of electrode size and shape can be realized, and multiple recording/stimulation sites can be fabricated on a single probe shank [9]. Starting with the pioneering work from Wise et al. [10], a growing number of silicon-based electrode arrays have been developed in the past and the performances of MEMS silicon microelectrodes have been improved in many aspects [11,12], e.g., three-dimensional arrays [13,14,15], dual-sided microelectrode arrays [16], integrated electronics or microfluidic channels [17,18,19,20,21,22], silicon probes for optical stimulation and imaging [23,24,25,26,27,28] or neurochemical signals detection [29,30,31]. In addition, one of the most important fabrication advancements is to integrate a greater number of recording sites on a single probe shank and simultaneously minimizing the probe geometry to avoid large tissue damages during the insertion. For example, Du et al. [32] designed and developed a high-density silicon-based neural probe employing 64-channel recording sites for performing large-scale, high-density electrophysiology in small, freely behaving animals. However, an inevitable consequence of reducing electrode size is that it will increase the electrode impedance, which introduces high Johnson noise during in vivo neural recording and therefore lowers the quality of recorded neural signals [33,34,35].

In order to solve the above-mentioned problems, many techniques have been proposed, and the most commonly used strategy is to modify small electrode sites with nanostructured materials, which greatly increase the surface area of electrode sites without increasing the size of electrode. In recent years, various nanomaterials have been used to reduce impedance of electrode sites, for example, Desai et al. [36] modified microwire multi-electrode arrays with platinum black to reduce electrode impedance and improve its in vivo neural recording performance. Keefer et al. [37] and Castagnola et al. [38] applied carbon nanotube to enhance neural recording. Other materials, such as iridium oxide [39,40], conducting polymers [41,42,43,44,45,46], gold nanoparticles [47,48], and nanocomposites [49,50,51,52,53] have also been applied to modify electrode sites. With the rapid development of material science, it is believed that more and more nanomaterials will be applied in this field.

In recent years, Au-Pt bimetallic nanoparticles have gained significant research attention and have been widely applied in a variety of fields, such as electrochemical sensors [54,55,56,57], fuel cells [58,59], and Raman scattering [60]. Compared with the corresponding monometallic nanoparticles (Au or Pt nanoparticles), Au-Pt bimetallic nanoparticles possess many favorable characteristics including increased surface area, improved electron transfer rate, enhanced electrocatalytic activity and promoted stability, which mainly result from the synergistic effects of these two monometallic nanoparticles. Recently, Kim et al. [61] electrochemically deposited Au-Pt bimetallic nanoparticles on gold multi-electrode arrays to improve its in vitro neuronal recording performance. However, it is well known that the surface morphology of nanoparticles plays a key role in their properties, and nanoparticles with different surface morphologies may possess different electrochemical characteristics [62]. In addition, electrochemical synthesis method has been widely used to prepare different kinds of metal nanoparticles due to its many advantages including simple synthesis and easily controlling the shape and size of nanoparticles [63]. Chemical dealloying method is often used to create nanoporous metals with tailored porosity [64,65]; for example, Kim et al. [66] applied porous Au films that were prepared via electro-deposition of Ag-Au alloy films followed by chemical dealloying of Ag to modify multi-electrode arrays for in vitro extracellular recording and stimulation. However, to the best of our knowledge, there is no report about the application of rough-surfaced AuPt alloy nanoparticles with nanoporosity prepared via electro-co-deposition of Au-Pt-Cu alloy nanoparticles followed by chemical dealloying Cu to modify gold neural microelectrode arrays.

In this paper, in order to improve the in vivo neural recording performance of our fabricated Michigan type silicon electrodes [67], we modified the round-shaped gold microelectrode sites (diameter of 20 μm) via electro-co-deposition of Au-Pt-Cu alloy nanoparticles followed by chemical dealloying Cu for the first time. Firstly, the prepared rough-surfaced Au-Pt alloy nanoparticles were characterized by scanning electron microscopy (SEM) and X-ray diffraction (XRD), which showed that the prepared AuPt alloy nanoparticles exhibited cauliflower-like shapes and possessed very rough surfaces with many different sizes of pores. Secondly, electrochemical impedance spectroscopy (EIS) was used to evaluate the impedance of modified microelectrode sites and results indicated that average impedance of rough-surfaced AuPt alloy nanoparticles modified sites was 0.23 MΩ at 1 kHz, which was only 4.7% of that of bare gold microelectrode sites (4.9 MΩ). Finally, thermal noises and in vivo spike recording performance of the microelectrode sites modified with rough-surfaced Au-Pt alloy nanoparticles were studied in details.

## 2. Materials and Methods

### 2.1. The Fabricated Microelectrode Arrays

**Figure 1:**
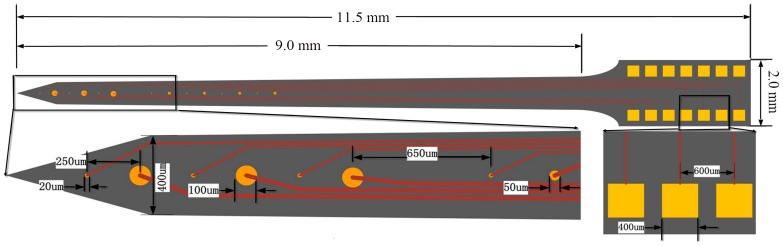
Schematic diagram of the designed microelectrode array.

The fabricated microelectrode array with dimensions of 9 mm × 400 μm × 200 μm (length × width × thickness) employed 14 round-shaped gold microelectrode sites distributed on each side. These sites could be divided into three types: three stimulating sites (diameter of 100 μm), seven electrophysiological recording sites (diameter of 20 μm) and four sites with diameter of 50 μm used for neurotransmitter measurements in future work. At the front of the designed microelectrode, three stimulating sites and three recording sites were mutually spaced with equal center-to-center distance of 250 μm. These four sites with diameter of 50 μm and the other four recording sites were also mutually spaced with equal center-to-center distance of 250 μm. Figure 1 showed the schematic diagram of the designed microelectrode array, and design and fabrication of the probes have been described in detail elsewhere [67]. In this paper, the in vivo neural recording and electrochemical performance of recording sites modified by rough-surfaced AuPt alloy nanoparticles were mainly studied. As for stimulating sites, their stimulating performance will be studied in future work.

### 2.2. Materials and Reagents

Chloroauric acid tetrahydrate (HAuCl_4_·4H_2_O) and chloroplatinic acid hexahydrate (H_2_PtCl_6_·6H_2_O) were purchased from Sangon Biotech. Co., Ltd. (Shanghai, China) and Aladdin Industrial Inc. (Shanghai, China), respectively. All other reagents were directly used without further purification. All aqueous solutions were prepared using ultrapure water (Millipore, ≥18 MΩ·cm, Billerica, MA, USA). The phosphate buffer solution (PBS, 0.1 mol/L) was prepared using 0.1 mol/L Na_2_HPO_4_ and 0.1 mol/L NaH_2_PO_4_ and the pH value of PBS was adjusted by mixing the stock solutions of NaH_2_PO_4_ and Na_2_HPO_4_ at different ratios.

### 2.3. Preparation of Rough-Surfaced Aupt Alloy Nanoparticles with Nanoporosity

Prior to preparation, the microelectrode arrays were sonicated in ethanol (1:1), HNO_3_ (1:1) and ultrapure water in sequence, and finally dried in air. Electrochemical co-deposition of Au-Pt-Cu alloy nanoparticles was carried out at room temperature in a conventional three-electrode electrochemical cell, which was composed of a gold recording site (diameter of 20 μm) of the microelectrode array as the working electrode, a platinum wire as the auxiliary electrode, and an Ag/AgCl (3 M KCl) as the reference electrode. Electrochemical co-deposition was performed at −0.25 V for about 600 s in 0.5 M H_2_SO_4_ solution containing 0.5 mM HAuCl_4_, 0.5 mM H_2_PtCl_6_ and 0.25 mM CuSO_4_. To get the rough-surfaced AuPt alloy nanoparticles with nanoporosity, the Cu was selectively dealloyed in 60% HNO_3_ at 70 °C for about one hour [64]. After dealloying, the microelectrode arrays were rinsed thoroughly with ultrapure water. For comparison, Au nanoparticles (AuNPs) modified gold recording sites via electrochemical deposition were prepared and used as a control along with the rough-surfaced AuPt alloy nanoparticles modified electrode sites.

### 2.4. Electrochemical and Physical Characterization

Electrochemical characterization of the microelectrode arrays were analyzed with the CHI 650E electrochemical analyzer (CH Instruments, Shanghai, China) at room temperature in a conventional three-electrode electrochemical cell, which was composed of a fabricated microelectrode array as the working electrode, a platinum wire as the auxiliary electrode, and an Ag/AgCl as the reference electrode. The electrochemical impedance spectroscopy (EIS) was performed in 0.1 M phosphate buffer solution (PBS) at an amplitude of 50 mV and frequencies between 1 Hz and 100 kHz.

Scanning electron microscopic images were collected on a Jsm-7800f field emission scanning electron microscopy (Electro Co., Tokyo, Japan). X-ray diffraction (XRD) patterns were carried out on an X’PertProPANalytical diffractometer using Cu Kα radiation (λ = 0.15418 nm).

### 2.5. In Vitro Background Noise Measurements

Background noise was measured to evaluate the relationship between electrode impedance and noise level. The background noise measurements were carried out in 0.9% NaCl solution and the background noise referenced to ground was recorded in a Faraday cage. The noise signals were recorded using a Cerebus multi-channel data acquisition system (Blackrock Microsystems, Salt Lake City, UT, USA) and then were filtered by a bandpass ranging from 1 Hz to 7.5 kHz and digitized (sampling rate: 30 kHz) for offline analysis. The noise signals were shown in time and frequency domains. Noise power spectra with frequency range of 1 to 1000 Hz were presented.

### 2.6. In Vivo Neural Recording and Data Analysis

In vivo neural recordings in the brain of the anesthetized rats were performed to compare the performance of recording spontaneous discharge signals at AuNPs and rough-surfaced AuPt alloy nanoparticles modified electrode sites. Male Sprague-Dawley (SD) rats weighing 250–300 g (Laboratory Animal Center at Xi’an Jiaotong University School of Medicine) were used in the electrophysiological experiments. All animal experiments were conducted in accordance with protocols approved by animal ethics committee of Xi’an Jiaotong University. The SD rats were anesthetized using an intraperitoneal injection of 2% pelltobarbitalum natricum solution (0.4 mL/100 g). After craniotomy and resection of the dura mater, the cortical surface was exposed.

For spike signals recording, the microelectrode array was implanted into lateral globus pallidus (GPe, AP: 1.4, ML: 3.4, DV: 5.5–6.5) of SD rat. Original signals were recorded at a sampling rate of 30 kHz and the recorded neural signals were filtered by a bandpass ranging from 500 Hz to 5 kHz to get the spike signals. Spike signals were analyzed offline using custom-automated MatLab (Mathworks Inc., Natick, MA, USA) software. The offline spike detection threshold is set at 5 σ, where σ is defined as [68]:

(1) $$\sigma \;=\;\mathrm{median}\left\{\frac{\left|\mathrm{spike}\;\mathrm{signals}\right|}{0.6745}\right\}$$

Note that taking a threshold based on the standard deviation of the signal (including the spikes) could lead to unreliable values, especially in cases with high firing rates and large spike amplitudes. In contrast, by using the threshold based on the median, the interference of the spikes is diminished [68]. The signal-to-noise ratio (SNR) was calculated as the root-mean-square (rms) of a spike divided by the standard deviation of the raw spike data trace [69].

## 3. Results and Discussion

### 3.1. Morphological and Structural Analysis

**Figure 2:**
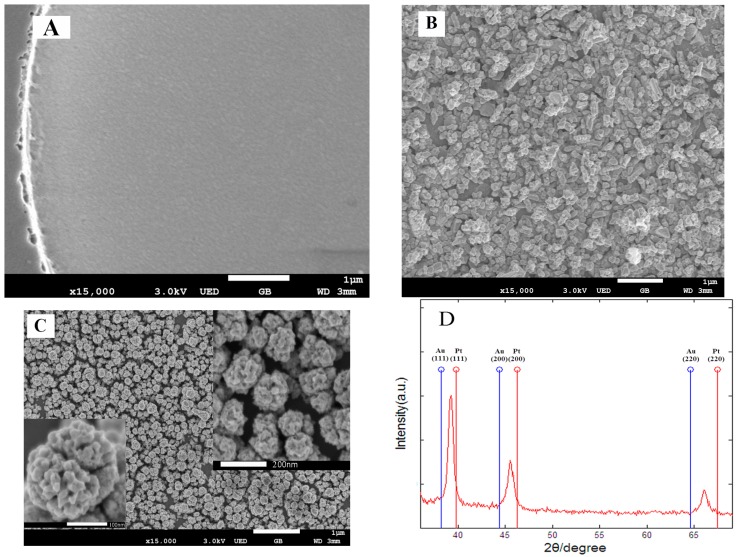
(**A**) Scanning electron microscopy (SEM) image of bare Au electrode site surface; (**B**) SEM image of Au nanoparticles (AuNPs); (**C**) SEM image of rough-surfaced AuPt alloy nanoparticles with nanoporosity; and (**D**) X-ray diffraction (XRD) patterns of the resulting rough-surfaced AuPt alloy nanoparticles.

The surface morphologies of the modified Au microelectrode sites were examined by scanning electron microscopy (SEM). As shown in Figure 2A, the bare Au microelectrode site possessed a smooth surface. After electrochemical deposition of AuNPs (Figure 2B), many gold nanoparticles with irregular shape were densely distributed on the surface of Au microelectrode site, which could increase the surface roughness and reduce impedance of electrode site. The surface morphology of rough-surfaced AuPt alloy nanoparticles with nanoporosity (Figure 2C) showed that the prepared AuPt alloy nanoparticles with a diameter of about 200 nm exhibited cauliflower-like shapes and were densely and uniformly distributed on the surface of Au microelectrode site. From the higher magnification SEM images (inset images of Figure 2C), the AuPt alloy nanoparticles possessed very rough surfaces and many different sizes of pores, which resulted from selective dealloying of Cu in Au-Pt-Cu alloy nanoparticles. Such nanostructures would greatly reduce microelectrode impedance, increase the surface roughness of microelectrode site, provide faster electron transfer rates and facilitate neural signal recording.

In order to further determine the feature of the obtained rough-surfaced AuPt alloy nanoparticles with nanoporosity, an XRD study was conducted. Figure 2D showed the XRD patterns of the obtained AuPt alloy nanoparticles. It was obvious that there appeared three well-resolved diffraction peaks, which could be assigned to the (111), (200) and (220) planes of AuPt alloy nanoparticles, respectively, demonstrating a face-centered cubic (fcc) structure. As is known, the peaks of pure gold nanoparticles (2θ = 38.2°, 44.4° and 64.6°) and pure platinum nanoparticles (2θ = 39.8°, 46.2° and 67.5°) are assigned to the (111), (200), and (220) planes, respectively. As shown in Figure 2D, the (111) peaks of rough-surfaced AuPt alloy nanoparticles fall well between (111) peaks of pure Au and Pt nanoparticles, suggesting that a single-phase alloy of AuPt rather than two separated phases of Pt and Au has been formed by electrochemical co-reduction of Au-Pt-Cu alloy nanoparticles followed by selective dealloying of Cu.

### 3.2. Electrochemical Behavior

**Figure 3:**
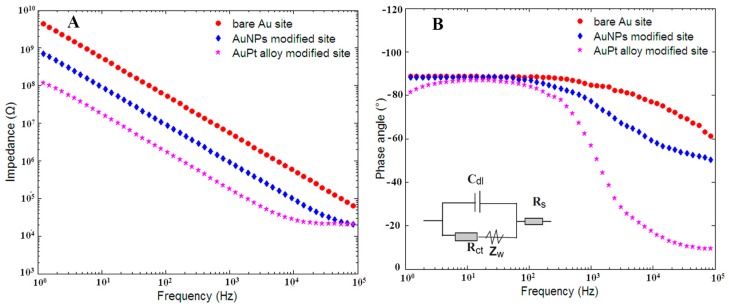
Results from electrochemical impedance spectroscopy (EIS) for: (**A**) the plot of interfacial impedances; and (**B**) the plot of phase angle for bare Au microelectrode site, AuNPs and rough-surfaced AuPt alloy nanoparticles modified gold micorelectrode sites. Inset of (**B**) shows Randles equivalent circuit.

Figure 3 showed the EIS of bare Au microelectrode site, AuNPs and rough-surfaced AuPt alloy nanoparticles modified Au micorelectrode sites. As can be seen in the Figure 3A, the impedances of these three microelectrode sites decreased as the frequency increased from 1 Hz and 100 kHz. The average impedance of bare Au microelectrode site was recorded as 4.9 MΩ, and after electro-deposition of AuNPs, the average impedance decreased to 0.9 MΩ, indicating that AuNPs could greatly increased the surface area and reduced electrode impedance. As for rough-surfaced AuPt alloy nanoparticles with nanoporosity modified microelectrode site, the average impedance further decreased to 0.23 MΩ, which was absolutely due to the very rough surfaces and many nanopores of AuPt alloy nanoparticles. In addition, for AuPt alloy nanoparticles, the synergistic effects of Au and Pt could provide more efficient electron transfer channels, faster electron transfer rates and better electric conductivity. As shown in the Figure 3B, the phase angle of bare Au microelectrode site at 1 kHz was ca. −84.7° and shifted to −77.3°, −56.6° for AuNPs and rough-surfaced AuPt alloy nanoparticles modified microelectrode sites, respectively, which showed that rough-surfaced AuPt alloy nanoparticles modified microelectrode site possessed more resistive electrode-electrolyte interface compared with other two microelectrode sites.

Inset of Figure 3B shows the Randles equivalent circuit of electrode-electrolyte interface and the corresponding three element values could be estimated using the following method. It is well known that this Randles equivalent circuit model includes a solution resistance (R_s_) and a double layer capacitor (C_dl_) in parallel with the series combination of a charge-transfer resistance (R_ct_) and Warburg impedance (W). The Warburg impedance W is generally negligible at high frequencies and in solutions with large amount of ions because the time interval is too short to be sufficient for the ion diffusion reacted on the electrode surface [35]. Therefore, the impedance can be simplified and expressed as:

(2) $$Z(\omega )=R_{S}+\frac{R_{\mathrm{ct}}}{1+j\omega R_{\mathrm{ct}}C_{\mathrm{dl}}}=Z^{\prime }\left(\omega \right)+{\mathrm{jZ}}^{\prime \prime }\left(\omega \right)$$

where ω is the angular frequency, $Z^{\prime }(\omega )$ is the real part of impedance and $Z^{\prime \prime }(\omega )$ is the imaginary part of impedance. Since charge transfer through the electrode-electrolyte interface was shunted by the double-layer capacitance at very high frequency, solution resistance R_s_ was determined as the asymptotic high frequency impedance:

(3) $$R_{s}=Z\left(f\to \infty \right)$$

and R_ct_ and C_dl_ at each frequency were determined as [70]:

(4) $$R_{\mathrm{ct}}(\omega )=\frac{Z^{\prime \prime 2}+{\left(Z^{\prime }-R_{S}\right)}^{2}}{Z^{\prime }-R_{S}}$$

(5) $$C_{\mathrm{dl}}(\omega )=-\frac{Z^{\prime \prime }}{\omega [{\left(Z^{\prime }-R_{S}\right)}^{2}+Z^{\prime \prime 2}]}$$

Based on the above-mentioned information, the values of solution resistance R_s_, double layer capacitor C_dl_ and charge-transfer resistance R_ct_ at 1 kHz of these three kinds of microelectrode sites could be estimated using the measured impedance data, and the obtained values were summarized in Table 1. As shown in Table 1, the measured solution resistance R_s_ of bare Au microelectrode site was about 12.1 KΩ, and it increased to 17.3 KΩ and 20.5 KΩ for AuNPs and rough-surfaced AuPt alloy nanoparticles modified microelectrode site, respectively. For round microelectrode site, the solution resistance R_s_ could be computed by using following equation [71,72]:

(6) $$\;\;\;\;\;\;\;R_{s}=\frac{\rho }{4r}$$

where ρ is the PBS solution resistivity (72 Ω·cm), r is the radius of microelectrode site. It is worth noting that $R_{s}\;$depends on the geometric area and is independent of the effective surface area of the microelectrode site. According to Equation (6), the theoretical value of $R_{s}\;$is 18 KΩ, which is reasonable compared with the measured values (12.1 KΩ, 17.3 KΩ and 20.5 KΩ). In addition, Table 1 shows that the significant reduction of impedance was mainly due to the combination of an increment of double-layer capacitances C_dl_ and a decrement in charge-transfer resistance R_ct_.

**Figure 4:**
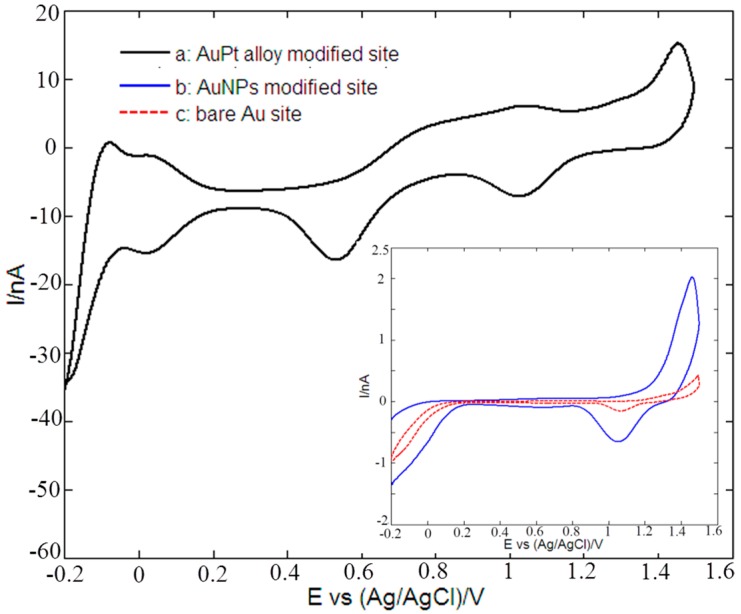
Cyclic voltammograms of: (**a**) bare Au microelectrode site; (**b**) AuNPs; and (**c**) rough-surfaced AuPt alloy nanoparticles modified gold micorelectrode sites in 0.5 M H_2_SO_4_ at scan rate of 50 mV/s.

Figure 4 shows the cyclic voltammetric responses of a bare Au microelectrode site (curve c), AuNPs (curve b) and rough-surfaced AuPt alloy nanoparticles (curve a) modified gold micorelectrode site in 0.5 M H_2_SO_4_. As for rough-surfaced AuPt alloy nanoparticles modified gold micorelectrode site (curve a), the potential peaks from −0.1 V to 0.2 V resulted from the hydrogen adsorption/desorption reactions. The reduction peaks at about 0.5 V and 1 V corresponded to the reduction of Au and Pt oxide species, respectively [73], which confirmed the presence of Au and Pt in the rough-surfaced AuPt alloy nanoparticles. As for bare Au microelectrode site (curve c), AuNPs modified site (curve b), the only one potential peak appearing at about 1 V corresponded to the reduction of Au oxide species. However, rough-surfaced AuPt alloy nanoparticles modified gold micorelectrode site showed almost the largest reduction peaks of Au and Pt oxide species in H_2_SO_4_ solution, which indicated that the modified microelectrode site possessed the highest metal electrochemical catalytic area.

**Figure 5:**
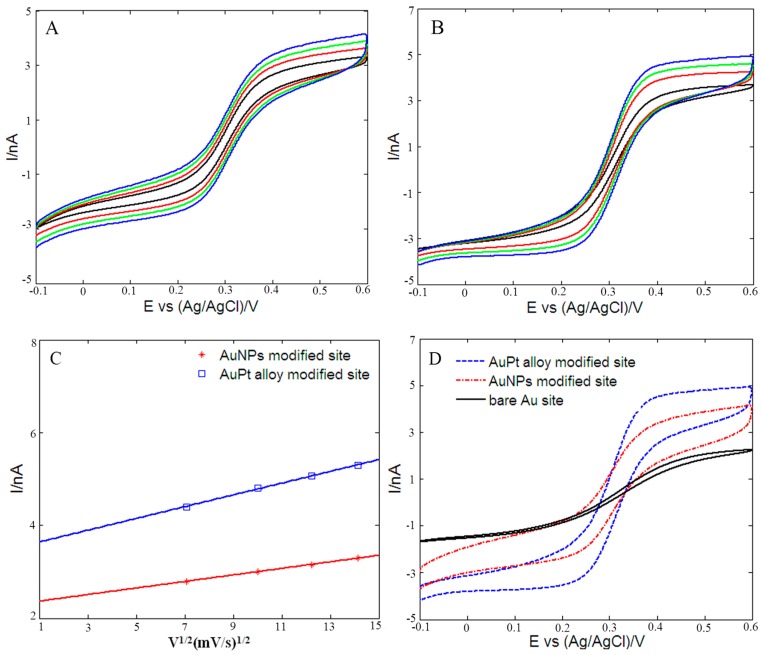
Cyclic voltammograms of: AuNPs (**A**); and rough-surfaced AuPt alloy nanoparticles (**B**) modified micorelectrode sites in 0.1 M KCl solution containing 2 mM [Fe(CN)_6_]^3−/4−^. Scan rate: 50, 100, 150 and 200 mV/s. (**C**) Plots of linear relationship between anodic peak currents ($I_{\mathrm{pa}}$) and square root of the scan rates. (**D**) Cyclic voltammograms of bare Au microelectrode site, AuNPs and rough-surfaced AuPt alloy nanoparticles modified micorelectrode sites in 2 mM [Fe(CN)_6_]^3−/4−^ at scan rate of 200 mV/s.

In order to further investigate the electrochemical properties of these three kinds of microelectrode sites, the cyclic voltammograms at different scan rates (50, 100, 150 and 200 mV/s) were obtained in 0.1 M KCl solution containing 2 mM [Fe(CN)_6_]^3−/4−^. Figure 5A,B showed the cyclic voltammograms of AuNPs and rough-surfaced AuPt alloy nanoparticles modified gold micorelectrode sites, respectively. For both AuNPs and rough-surfaced AuPt alloy nanoparticles modified gold micorelectrode sites, the absolute values of reduction and oxidation peak currents were almost equal, and the peak potentials were definite values and independent of the scan rates, which indicated that the electrochemical processes of the modified microelectrode sites in 0.1 M KCl solution containing 2 mM [Fe(CN)_6_]^3−/4−^ were reversible. In addition, the values of the peak currents increased with scan rates varying from 50 mV/s to 200 mV/s, and reduction and oxidation peak currents of the modified microelectrode sites were proportional to the square root of the scan rates in the range 50–200 mV/s (Figure 5C), suggesting the electrochemical responses were a typical surface-controlled process [74]. The electro-active surface area of microelectrode sites can be estimated for a reversible and diffusion controllable process according to the Randles–Sevcik equation [75,76]:

(7) $$I_{p}=2.69\times 10^{5}{A\cdot D}^{\frac{1}{2}}\cdot n^{\frac{3}{2}}\cdot v^{\frac{1}{2}}\cdot C$$

where $I_{p}$ is the redox peak current, n is the the electron number involved in oxidation reaction, and, here, n is equal to 1 for [Fe(CN)_6_]^3−/4−^ redox system. C represents the concentration (mol·cm^−3^) of the ferricyanide (here, C = 2$\;\times 10^{-3}$), and D is the diffusion coefficient of the molecule in solution (cm^2^·s^−1^), which is 6.7 $\times 10^{-6}$ at 25 °C. v is the scan rate (V·s^−1^) and A represents the electro-active surface area of microelectrode sites.

According to the linear relationship between $I_{\mathrm{pa}}$ and v^1/2^ (Figure 5C), the values of the electro-active surface area were 1579.8 μm^2^ for AuNPs modified site and 2871.2 μm^2^ for rough-surfaced AuPt alloy nanoparticles modified micorelectrode site. For bare Au microelectrode site (round-shaped, diameter of 20 μm), the geometric area is 314.2 μm^2^. Therefore, electro-active surface area of rough-surfaced AuPt alloy nanoparticles modified micorelectrode site is almost 9.1 times as large as that of bare gold microelectrode site, showing that rough-surfaced AuPt alloy nanoparticles modified micorelectrode site possessed a very rough surface. Figure 5D showed the cyclic voltammograms of these three microelectrode sites in the same 2 mM [Fe(CN)_6_]^3−/4−^ at scan rate of 200 mV/s. It was obvious that bare Au microelectrode site exhibited small redox peak currents, and after deposition of AuNPs, the redox peak currents increased significantly. As for rough-surfaced AuPt alloy nanoparticles modified micorelectrode site, the redox peak currents further increased, indicating that the order of redox peak current was consistent with the above-calculated electro-active surface area, i.e., bare Au microelectrode site < Au NPs modified microelectrode site < AuPt alloy nanoparticles modified microelectrode site. These results implied that the rough-surfaced AuPt alloy nanoparticles modified micorelectrode site possessed bigger electro-active surface areas and higher conductivity compared with other two microelectrode sites.

### 3.3. Background Noise Analysis

In vitro background noises of microelectrode sites included the electronic noise due to the amplifier, the thermal noise and a number of noise sources associated with the double layer interface between solution and microelectrode surface [77]. Thermal noise was generated by the thermal agitation of electrons inside an electrical conductor at equilibrium, and it was considered as one of the main noise sources for microelectrode site recordings [36]. Thermal noise could be computed using the following equation [77]:

(8) $$V_{\mathrm{rms}}=\sqrt{{k\cdot T\cdot Z}_{\mathrm{real}}\cdot ∆f}$$

where $V_{\mathrm{rms}}$ is the thermal noise in the form of root mean square, k is the Boltzmann constant, T is the absolute temperature, $Z_{\mathrm{real}}$ is the average of the measured real part of the component of the complex impedance and ∆f is the bandwidth over which the noise is measured. Therefore, noise is proportional to the square root of the impedance of the electrode and decreasing the impedance of the microelectrode sites should lower the thermal noise.

**Figure 6:**
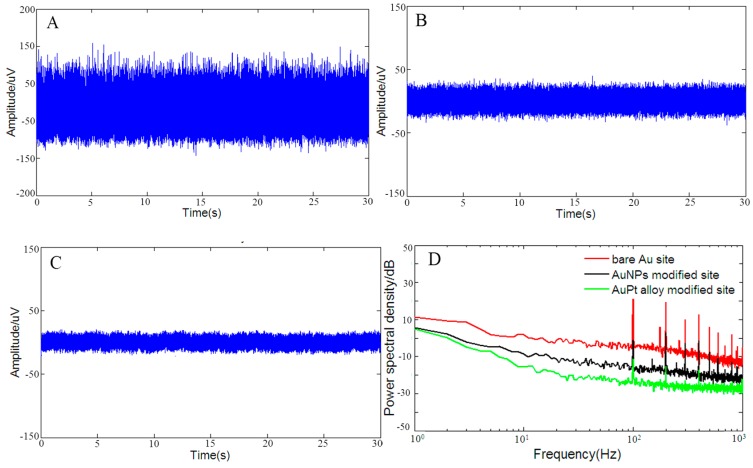
In vitro background noise varying from 1 to 7500 Hz of: bare Au microelectrode site (**A**); AuNPs (**B**); and rough-surfaced AuPt alloy nanoparticles (**C**) modified micorelectrode sites. (**D**) Noise power spectral density (PSD) from 1 to 1000 Hz. The 50 Hz power-line interference was filtered from the noise signals.

Figure 6 shows the in vitro background noises recorded in 0.1 M PBS of bare Au microelectrode site (Figure 6A), AuNPs (Figure 6B) and rough-surfaced AuPt alloy nanoparticles (Figure 6C) modified micorelectrode sites. Here, noise level was computed in the form of root mean square $V_{\mathrm{rms}}$, which was defined as $V_{\mathrm{rms}}=\sqrt{\frac{\sum^{}y_{k}^{2}}{K}}$, where $y_{k}$ (*k* = 1 − K, K = 60,000, i.e., two seconds data points) was the noise amplitude at sampling point *k*. Therefore, the measured $V_{\mathrm{rms}}$ in the the frequency band of 1–7500 Hz was 34.1${\;\mu V}_{\mathrm{rms}}$, 11.4 ${\mu V}_{\mathrm{rms}}$ and 7.5 ${\mu V}_{\mathrm{rms}}$ for bare Au microelectrode site, AuNPs and rough-surfaced AuPt alloy nanoparticles modified micorelectrode sites, respectively, which indicated that noise of rough-surfaced AuPt alloy nanoparticles modified micorelectrode site was lowered to only 21.9% of that of bare Au microelectrode site. As shown in frequency domain (Figure 6D), it was obvious that rough-surfaced AuPt alloy nanoparticles modified micorelectrode site possessed the lowest average noise power in the frequency band of 1–1000 Hz. Based on Equation (8), the calculated noise level from 1 to 7500 Hz was 19.8${\;\mu V}_{\mathrm{rms}}$, 6.1 ${\mu V}_{\mathrm{rms}}\;$and 4.7 ${\mu V}_{\mathrm{rms}}$ for bare Au microelectrode site, AuNPs and rough-surfaced AuPt alloy nanoparticles modified micorelectrode sites, respectively, which showed that the measured noise were reasonable compared to the calculated noises and thermal noise decreased with the increase of microelectrode surface area.

Márton et al. [78] pointed out that the in vitro and in vivo electrochemical impedance were different, and the reason is that impedance depends not only on electrode, but on solution properties as well [79,80]. As for 0.1 M PBS solution, the resistivity is 72 Ω·cm, but the average resistivity of gray mattey is 4.11 Ω·m (411 Ω·cm) according to a review study [81]. Although the data of in vivo electrochemical impedance are not measured, we can compare in vitro solution resistance R_s_ and in vivo solution resistance R_s_ according to Equation (6). The computed in vitro and in vivo R_s_ of AuPt alloy modified recording microelectrodes is 20.5 KΩ and 117 KΩ, respectively. Even though we did not obtain the data of in vivo electrochemical impedance, it can be inferred that the in vivo impedance of AuPt alloy modified microelectrodes is bigger than our measured in vitro impedance, which will result in a certain increase of in vivo thermal noise. However, as for in vivo extracellular recording, the recorded neural signals are affected by biological and non-biological noise sources [77]. The non-biological noise sources include the electronic noise of amplifier, the thermal noise and some noise sources associated with double layer interface between solution and microelectrode surface. The biological noise sources include: (a) the additional thermal noise component due to the presence of brain tissue, which increases the electrode impedance real part; and (b) small neural signals, emitted by distant neurons/neuronal processes too far to be detected as single units and therefore considered as noise for most neural signal analyses. In summary, the background noise components of recorded in vivo neural signals are complex. Here, we do not want to focus on analysis of in vivo background noise components, alternatively, it is helpful to use e.g., signal-to-noise ratio (SNR) to evaluate in vivo extracellular recording performance of AuPt alloy modified recording microelectrodes. Therefore, in the next section, the in vivo spike signal recordings were carried out.

### 3.4. Improvement of In Vivo Extracellular Recording

On the one hand, it was necessary to confirm that the rough-surfaced AuPt alloy nanoparticles increased the surface area of microelectrode sites without compromising single-unit selectivity. On the other hand, it might be interesting to explore whether rough-surfaced AuPt alloy nanoparticles modified microelectrode sites improved performance of in vivo spike signal recordings. Therefore, in vivo spike signals recording was performed in SD rats. It was obvious that bare Au sites were not suitable for in vivo spike signal recording due to its high impedance (4.9 MΩ), therefore, we only carried out spike signals recordings at AuNPs and rough-surfaced AuPt alloy nanoparticles modified microelectrode sites. Five AuNPs and five rough-surfaced AuPt alloy nanoparticles modified microelectrode sites were used to perform ten times in vivo spike signal recordings, respectively.

**Figure 7:**
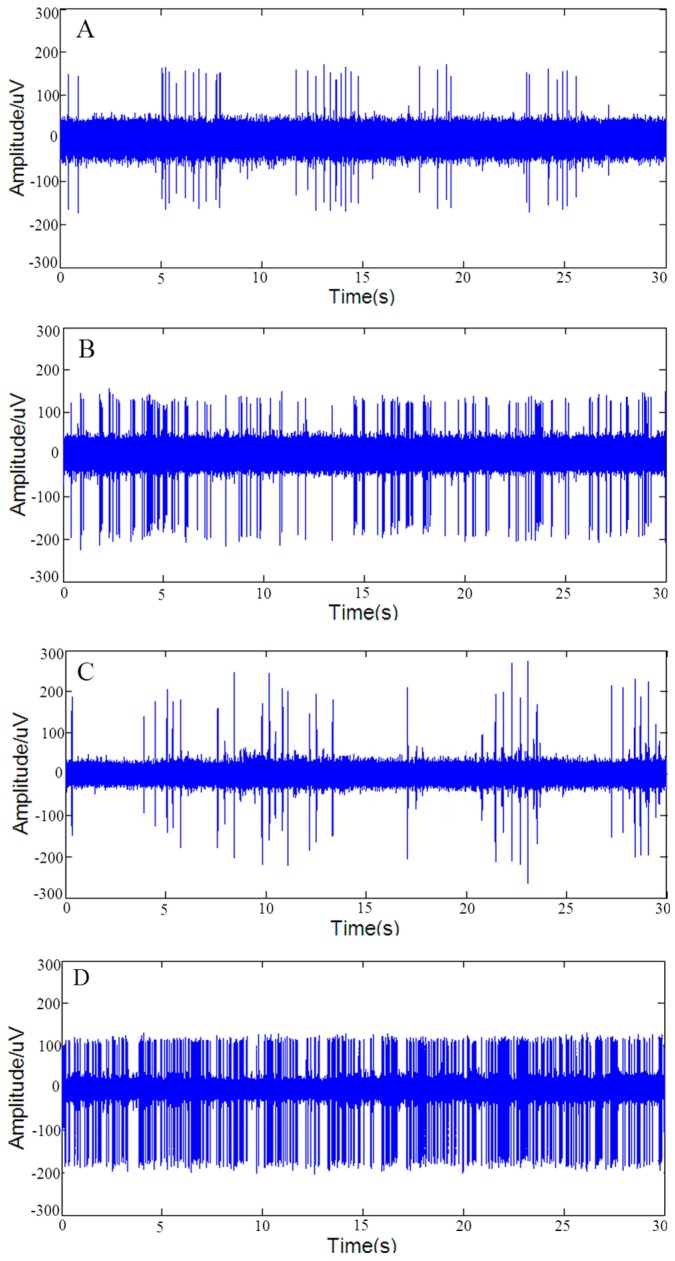
In vivo spontaneous spike signals and sorted spikes of AuNPs (**A**,**B**); and rough-surfaced AuPt alloy nanoparticles (**C**,**D**) modified micorelectrode sites.

Figure 7 shows typical segments of spontaneous spike signals and sorted spikes recorded at AuNPs (Figure 7A,B) and rough-surfaced AuPt alloy nanoparticles (Figure 7C,D) modified microelectrode site, respectively. As shown in Figure 7, two kinds of firing patterns of lateral globus pallidus (GPe) neurons [82], i.e., high frequency firing with pause (Figure 7B,D) and low frequency firing with pause (Figure 7A,C) were detected at both modified Au sites. In addition, the average spike amplitude and average SNR of both modified microelectrode sites were calculated and shown in Table 2. As shown in Table 2, the average spike amplitude of AuPt alloy nanoparticles modified sites was similar with that of AuNPs modified sites, but the average SNR was obviously higher than that of AuNPs modified sites, which was mainly due to the reduction of in vivo recording background noise at AuPt alloy nanoparticles modified sites. The reduction of in vivo recording background noise at AuPt alloy nanoparticles modified sites might be due to reduction of impedance (Figure 3) and resulting decrease of thermal noise (Figure 6). The impedance of electrodes at 1 kHz was thought to affect the SNR of spike recordings because spike waveforms were usually around this frequency. The above results indicated that the rough-surfaced AuPt alloy nanoparticles not only increased the surface area of microelectrode sites without compromising single-unit selectivity, but also improved SNR of in vivo spike signals.

Comparison of microelectrode properties with other modified microelectrodes reported in the literature is summarized and shown in Table 3. As shown in Table 3, SNR at rough-surfaced AuPt alloy nanoparticles modified sites is comparable to those of surfactant-templated ordered PEDOT [45] and PEDOT-PSS composite/Poly(p-xylylene) [34] modified sites, but is bigger than those of PEDOT [38] and PEDOT/pTS composite [42] modified sites. In summary, rough-surfaced AuPt alloy nanoparticles modified microelectrode sites exhibited relatively high average spike SNR. The impedance at 1 kHz is smaller than those of PEDOT [38] and GaP nanowires [83] modified sites, but is bigger than those of other microelectrodes. However, impedance is closely related to the size of microelectrode site. Because the sizes of microelectrode sites reported in the literatures are different, so it is not convenient to compare their impedance values.

As for Reference [61], the authors also used AuPt alloy nanoparticles to modify microelectrode sites, but the preparation method is different from ours. The authors in Reference [61] electrochemically deposited AuPt bimetallic nanoparticles on gold multi-electrode arrays to improve its in vitro neuronal recording performance. However, we modified the Michigan type silicon electrodes via electro-co-deposition of Au-Pt-Cu alloy nanoparticles followed by chemical dealloying Cu, and applied it for in vivo neuronal recordings. It is well known that the surface morphology of nanoparticles plays a key role in their properties, and nanoparticles with different surface morphologies may possess different electrochemical characteristics. As for AuPt nanoparticles modified sites [61], its impedance at 1 kHz is 6.67% of that of bare sites, i.e., the impedance of the bare sites decreased from 6 × 105 Ω (at 1 kHz) to 4.0 × 104 Ω (at 1 kHz) upon electro-deposition of AuPt nanoparticles. However, for our prepared rough-surfaced AuPt alloy nanoparticles modified sites, its impedance at 1 kHz is 0.23 MΩ, which is only 4.7% of that of bare microelectrode sites (4.9 MΩ). Therefore, although absolute impedance value of rough-surfaced AuPt alloy nanoparticles modified sites is bigger than that of AuPt nanoparticles modified sites, we believe that rough-surfaced AuPt alloy nanoparticles possessed faster electron transfer rate than AuPt nanoparticles modified sites.

### 3.5. Durability Test

**Figure 8:**
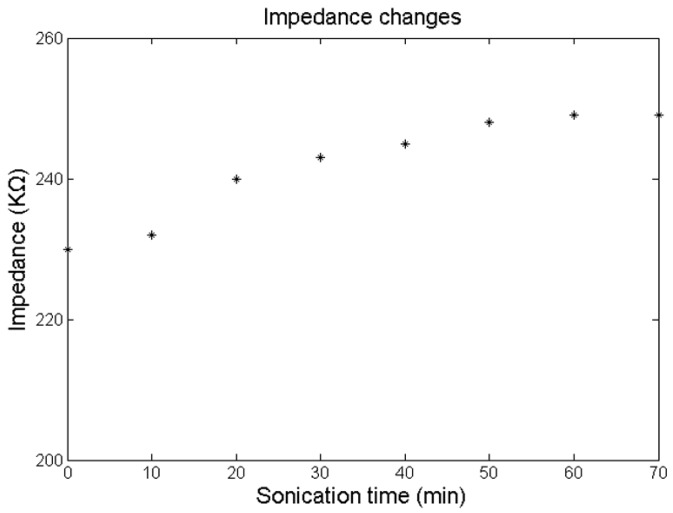
Impedance changes (at 1 kHz) of rough-surfaced AuPt alloy nanoparticles modified microelectrode arrays during ultrasonic treatment.

To confirm the mechanical durability of the prepared rough-surfaced AuPt alloy nanoparticles, the as-prepared rough-surfaced AuPt alloy nanoparticles modified microelectrode arrays were sonicated in PBS seven times (ten min each time) and after each sonication microelectrode impedance was tested. Figure 8 showed the impedance changes (at 1 kHz) during ultrasonic treatment. The initial average impedance (at 1 kHz) of rough-surfaced AuPt alloy nanoparticles modified microelectrode arrays was 230 KΩ (0.23 MΩ) and after seven times of ultrasonic treatment the average impedance (at 1 kHz) increased to 249 KΩ, which showed an increase of only 8.3% of initial impedance. These results confirmed the mechanical durability of rough-surfaced AuPt alloy nanoparticles modified microelectrode arrays.

## 4. Conclusions

In this paper, rough-surfaced AuPt alloy nanoparticles with nanoporosity were deposited on gold microelectrode sites through electro-co-deposition of Au-Pt-Cu alloy nanoparticles, followed by chemical dealloying Cu. Both in vitro and in vivo methods including SEM, EIS, CV and in vivo neural recording were used to evaluate the performance of the modified microelectrode sites. In conclusion, rough-surfaced AuPt alloy nanoparticles could greatly increase the effective surface area and reduce the impedance of microelectrodes, which leaded to reduction of thermal noise and improvement of in vivo spike recordings. The origin of impedance reduction was studied by using a simple equivalent circuit and the results showed that the significant reduction of impedance was mainly due to the combination of an increment of double-layer capacitance and a decrement in charge-transfer resistance. In short, this paper proposed a convenient way for reducing impedance of microelectrode sites and improving the quality of in vivo spike recordings.

## Figures and Tables

**Table 1:** Element values of Randles equivalent circuit at 1 KHz for three kinds of microelectrode sites.

Microelectrode Site	R_s_ (KΩ)	R_ct_ (MΩ)	C_dl_ (μF)
bare Au microelectrode site	12.1	61	0.12
AuNPs modified site	17.3	4.6	0.78
AuPt alloy modified site	20.5	0.45	4.02

**Table 2:** Comparison of microelectrode properties between AuNPs and rough-surfaced AuPt alloy nanoparticles modified sites.

	AuNPs Modified Site	AuPt Alloy Modified Site
Average impedance at 1 kHz (MΩ)	0.9	0.23
Average spike amplitude (μV)	281.2	273.7
Average SNR	3.4	4.8

Note: AuNPs stands for Au nanoparticles; SNR stands for signal-to-noise ratio.

**Table 3:** Comparison of microelectrode properties with other modified microelectrodes reported in the literatures.

Microelectrode Modified Materials	Deposition Techniques	Spike SNR	Impedance at 1 kHz (KΩ)	References
Platinum black	Ultrasonic electroplating	-	5	[36]
CNT/gold composite	Electrochemical deposition	-	38	[37]
PEDOT	Electrochemical deposition	~1.6	370	[38]
PEDOT/pTS composite	Electrochemical deposition	4.1	35	[42]
Surfactant-templated ordered PEDOT	Electrochemical deposition	5.1	130	[45]
PEDOT-PSS composite/Poly(p-xylylene)	CVD/electrochemical deposition	4.7	~60	[34]
PEDOT-CNT composite	Electro-polymerization	-	15	[40]
GaP nanowires	MOVPE	-	1200	[83]
Platinum black	Electrochemical deposition	-	38	[78]
Platinum nanoparticles	Electrophoretic deposition	-	~40	[84]
AuPt nanoparticles	Electro-co-deposition	-	40	[61]
Rough-surfaced AuPt alloy nanoparticles	Electro-co-deposition/chemical dealloying	4.8	230	This work

Note: CNT stands for carbon nanotube; PEDOT stands for Poly(3,4-ethylenedioxythiophene); pTS stands for para-toluene sulfonate; PSS stands for poly(styrenesulphonate); CVD stands for chemical vapor deposition; CNT stands for carbon nanotube; GaP stands for gallium phosphide; MOVPE stands for metalorganic vapor phase epitaxy.
